# Tumor protease-activated theranostic nanoparticles for MRI-guided glioblastoma therapy

**DOI:** 10.7150/thno.79342

**Published:** 2023-03-13

**Authors:** Ching-Hsin Huang, Edwin Chang, Li Zheng, Joe Gerald Jesu Raj, Wei Wu, Laura J. Pisani, Heike E. Daldrup-Link

**Affiliations:** 1 Department of Radiology, Molecular Imaging Program at Stanford (MIPS), Stanford University, CA, U.S.A.; 2 Stanford Center for Innovation in In vivo Imaging (SCi 3 ) at Porter, Canary Center for Cancer Early Detection, Stanford University, CA, U.S.A.; 3 Sarafan Chemistry, Engineering & Medicine for Human Health (Chem-H), Stanford University, Stanford, CA, U.S.A.; 4 Stanford Center for Innovation in In vivo Imaging (SCi 3 ) at Clark, James H. Clark Center, Stanford University, CA, U.S.A.

**Keywords:** glioblastoma, theranostic, nanoparticles, MMAE, MRI

## Abstract

**Rationale:** As a cancer, Glioblastoma (GBM) is a highly lethal and difficult-to-treat. With the aim of improving therapies to GBM, we developed novel and target-specific theranostic nanoparticles (TNPs) that can be selectively cleaved by cathepsin B (Cat B) to release the potent toxin monomethyl auristatin E (MMAE).

**Methods:** We synthesized TNPs composed of a ferumoxytol-based nanoparticle carrier and a peptide prodrug with a Cat-B-responsive linker and the tubulin inhibitor MMAE. We hypothesized that intratumoral Cat B can cleave our TNPs and release MMAE to kill GBM cells. The ferumoxytol core enables *in vivo* drug tracking with magnetic resonance imaging (MRI). We incubated U87-MG GBM cells with TNPs or ferumoxytol and evaluated the TNP content in the cells with transmission electron microscopy and Prussian blue staining. In addition, we stereotaxically implanted 6- to 8-week-old nude mice with U87-MG with U87-MG GBM cells that express a fusion protein of Green Fluorescence Protein and firefly Luciferase (U87-MG/GFP-fLuc). We then treated the animals with an intravenous dose of TNPs (25 mg/kg of ferumoxytol, 0.3 mg/kg of MMAE) or control. We also evaluated the combination of TNP treatment with radiation therapy. We performed MRI before and after TNP injection. We compared the results for tumor and normal brain tissue between the TNP and control groups. We also monitored tumor growth for a period of 21 days.

**Results:** We successfully synthesized TNPs with a hydrodynamic size of 41 ± 5 nm and a zeta potential of 6 ± 3 mV. TNP-treated cells demonstrated a significantly higher iron content than ferumoxytol-treated cells (98 ± 1% vs. 3 ± 1% of cells were iron-positive, respectively). We also found significantly fewer live attached cells in the TNP-treated group (3.8 ± 2.0 px^2^) than in the ferumoxytol-treated group (80.0 ± 14.5 px^2^, p < 0001). *In vivo* MRI studies demonstrated a decline in the tumor signal after TNP (T_2_= 28 ms) but not control (T_2_= 32 ms) injections. When TNP injection was combined with radiation therapy, the tumor signals dropped further (T_2_ = 24 ms). The combination therapy of radiation therapy and TNPs extended the median survival from 14.5 days for the control group to 45 days for the combination therapy group.

**Conclusion:** The new cleavable TNPs reported in this work accumulate in GBM, cause tumor cell death, and have synergistic effects with radiation therapy.

## Introduction

Glioblastoma (GBM) is the deadliest malignant primary brain tumor in humans, with a median survival time of less than one year 1,2. Despite available treatment options of aggressive surgery and radio/chemotherapy, tumor recurrence is common 1,3,4. Major challenges in achieving effective GBM therapy responses are tumor-specific drug delivery and off-target side effects. Preclinical and clinical studies have investigated potent tubulin polymerization inhibitors from the monomethyl auristatin family conjugated to antibodies (known as antibody-drug conjugates or ADCs) as potential treatments for a wide variety of cancers including GBM 5,6, 22. This ADC approach can successfully deliver cytotoxic drugs but often leads to inadequate retention in tumors due to substantial intertumoral and intratumoral variation in antibody accumulation 23, 24. Antibodies in ADCs may also cause nephrotoxicity 14. Moreover, in a GBM U87ΔEGFR-luc xenograft model, the heterogeneous drug/antibody ratio failed to reach a satisfying therapeutic outcome 14, 25. As an alternative to antibodies, researchers have developed nanoparticles with reductive-sensitive linkers or prostate-specific membrane antigen (PSMA)-targeting plus cathepsin B (Cat B)-activatable linkers to deliver tubulin inhibitors to tumors 5,7.

The lack of imaging monitoring at the accumulation site of therapeutic agents also causes difficulties for GBM therapy. Although nanoparticle conjugates have demonstrated substantially higher drug loading compared to ADCs, the current nanoparticles cannot be tracked by magnetic resonance imaging (MRI), the most common imaging modality in GBM. Levels of both glutathione, which modulates the reductive-sensitive drug release, as well as PSMA, which targets the tumors, are highly varied between GBM patients and throughout the disease's course. This makes it important to actively monitor the drug *in vivo* 8,9. Thus, a theranostic strategy that can successfully deliver therapeutic agents to brain tumors and provide *in vivo* tracking of drug accumulation is needed for GBM treatment.

To be able to track a drug within the brain and avoid toxic side effects, we designed non-toxic peptide prodrug nanoparticles that are activated by specific tumor enzymes which are overexpressed in brain cancers. Cat B is highly overexpressed in GBM (present in >99% of GBM tumors but absent in the normal brain 10,11) and is a good target for prodrug activation due to its proteolytic nature that selectively cleaves specific peptide sequences 12. Cat B is vital for glioma cell growth, invasion, and angiogenesis. We attached the peptide prodrug, including the potent tubulin inhibitor MMAE and a Cat B-cleavable linker, to the amine-modified nanoparticle compound ferumoxytol. This modification improved tumor delivery, tumor enzyme activation, and therapeutic efficacy compared with the original small molecule, MMAE. Ferumoxytol nanoparticles are Food and Drug Administration (FDA) approved for intravenous (i.v.) treatment of anemia in patients and have been successfully used in tumor imaging 13,14. Thus, our nanoparticle conjugated drug is clinically translatable. In addition, the ferumoxytol nanoparticle backbone can be detected by MRI, thereby enabling *in vivo* drug tracking. Here, we evaluated whether these dual therapeutic and diagnostic (i.e. theranostic) nanoparticles (TNPs) can provide: 1) tubulin-inhibitory-mediated tumor disruption, 2) drug delivery to GBM, and 3) significant therapeutic efficacy in rodent GBM models.

## Materials and Methods

### Synthesis of TNPs

Ferumoxytol was run through a column filled with Sephadex G-25 (Fisher Scientific) and re-constituted in 0.1 M MES sodium salt (Sigma) buffer at pH = 6-6.4. Amicon Ultra-15® centrifuge filters (10 KDa cutoff, 2,000× g, 20 min, 4 °C) were used for elution and sample concentration. The process of MES buffer addition and centrifugation was repeated three times. The concentrated samples were reacted with anhydrous hydroxybenzotriazole (HOBt, ApexBio) and 1-ethyl-3-(3-dimethylaminopropyl)carbodiimide (EDC, Fisher Scientific) at a molar ratio of Fe:HOBt:EDC = 2.5:0.8:3 in a shaker at 37 °C for 20 min. Then, 1,2-diaminoethane (Sigma Aldrich) was added and reacted in a 50 °C water bath for 90 min. Samples were vortexed in between. Samples were washed with MES buffer using Amicon Ultra-15® centrifuge filters three times and dialyzed against MES buffer overnight at 4 °C. The next day, particles were grafted with peptide prodrugs (cathepsin-cleavable MMAE, C-MMAE) that contain four poly(ethylene glycol) linkers, cleavable valine-citrulline dipeptide, a p-aminobenzylcarbamate spacer, and MMAE toxin at its N-hydroxysuccinide ester moiety (BroadPharm, San Diego). The ratio among iron oxide nanoparticles and amine functionalization and prodrug grafting concentration were based on the previous ferumoxytol-based modification protocol 15. Functionalized particles and C-MMAE were mixed in a 37 °C shaker for 3 h. Grafted ferumoxytol nanoparticles were washed and concentrated with Amicon Ultra-15® centrifuge filters and underwent dialysis against phosphate buffered saline (PBS) overnight to yield TNPs. Purification with Amicon Ultra-15® centrifuge filters was repeated until the filtrate had no detectable C-MMAE, yielding the final TNP product. The MMAE release from purified TNP co-incubated with cathepsin B enzyme was measured by LC/MS-MS (**Figure 2A**) to ensure the success of conjugation.

### Characterization of TNPs

Nanoparticles were examined by a transmission electron microscope (TEM; JEOL JEM1400) on formvar/carbon supported copper grids (Sigma Aldrich; size 200 mesh). The iron concentrations were measured by an inductively coupled plasma optical emission spectrometer (ICP-OES) on a Thermo ICAP 6300 Duo View Spectrometer with a solid-state CID detector. Quantification of the free prodrug C-MMAE was performed using ultra high-performance liquid chromatography (UHPLC) on a Dionex HPLC system. The system was equipped with a GP50 gradient pump and an inline diode array UV-Vis detector. A C12 column (Phenomenax, Jupiter 4u Proteo 90A, 4 μm, 4.6 × 250 mm) was used with a gradient mobile phase of acetonitrile (B)/H2O (A) containing 0.1% trifluoroacetic acid (TFA). Quantification of free MMAE was performed by Agilent 6470 triple quadrupole with an Agilent 1290 Infinity II UHPLC with a C8 column (2.1 x 50 mm, 1.8 µm) (Agilent, Santa Clara) with multiple reaction monitoring (MRM) at product ion fragments of 152.1 and 686.6. The amount of MMAE on nanoparticles was calculated by the difference in MMAE in the reaction solutions before and after conjugation. Nanoparticles were suspended in deuterium oxide (D_2_O, MagniSolv) at 0.25-60 mM concentrations for nuclear magnetic resonance (NMR) measurement. Relaxation time 1 (T1), relaxation time 2 (T2), relaxivity 1 (r1), and relaxivity 2 (r2) were obtained from Varian Inova 500 MHz NMR with a VNMRJ 4.2 using a 5 mm 5 mm X{H} probe at 25 °C. The hydrodynamic diameter, polydispersity index, and zeta-potential of nanoparticles were measured by a NanoBrook Omni analyzer (BrookHaven Instruments).

### Cell Culture

U87-MG human GBM cell lines were used for *in vitro* and *in vivo* studies. U87-MG cells were kindly provided by Dr. Edwin Chang at the Stanford Center for Innovation in *In vivo* Imaging (SCi^3^) at Porter, Canary Center. U87-MG cells were grown in Dulbecco's Modified Eagle Medium (Life Technologies) containing 10% fetal bovine serum and 1% penicillin/streptomycin (Life Technologies). Cat B expression of GBM cell lines was assessed by intracellular staining analysis of paraformaldehyde-fixed and methanol-permeabilized samples. Cat B (D1C7Y) XP® rabbit monoclonal antibody (31718, Cell Signaling) and anti-rabbit Alexa Fluor® 647 conjugated antibody (A21245, Invitrogen) were used for staining.

### *In vitro* evaluation of the therapeutic efficacy of TNP against GBM cancer cells

U87-MG cells were plated in 96-well plates at 3 × 10^4^ cells/well and incubated overnight at 37 °C to allow for cellular adherence. Concentrations ranging from 0.001 nM to 100 nM of MMAE, C-MMAE, TNP, and ferumoxytol in PBS were added. Cells were incubated at 37 °C with 5% CO_2_ for 72 h. The amount of nanoparticles in the GBM cells was observed by recording the absorbance peak at 450 nm (peak of ferumoxytol) by a microplate reader (Expert Plus V1.4 ASYS). For the cell uptake study, Alexa Fluor 555 dye conjugated MMAE and C-MMAE (Fisher Scientific) were used to visualize drugs within GBM cells. Cell samples were stained with Prussian blue (Sigma) for ferumoxytol visualization. Cell viability was assessed by cell adherence following addition of 0.2% of hexamethyl pararosaniline chloride (crystal violet, Acros Organics) as well as examination of the alive and adherent cells under microscopy. Cell viability dependency on drug concentration was also verified using a Cell Titer Blue assay kit (Promega) as per the manufacturer's instructions. At the end of the incubation period, the fluorescence was measured at excitation/emission 550/585 nm in a microplate reader (Expert Plus V1.4 ASYS). Data were normalized relative to the vehicle-treated controls. For observing TNPs within GBM cells, 7 mm × 7 mm plastic slices were placed in 24-well plates, on top of which U87-MG cells were seeded at a density of 1.8 × 10^5^ cells/well. TNPs (4 nM) were added and incubated at 37 °C, 5% CO_2_ for up to 120 min. Plastic slices were collected from naïve control and TNP-treated U87-MG cells and fixed and embedded for TEM observation (JEOL JEM1400).

### *In vivo* evaluation of the therapeutic efficacy of TNP against GBM cancer cells in an orthotopic intracranial xenograft model

A total of 4 × 10^5^ U87-MG/luciferase/green fluorescent protein (GFP) cells were injected stereotaxically into the striatum of anesthetized 6‐ to 8‐week‐old female NU/J homozygous nude mice (Jackson Laboratory), using the following coordinates: 2 mm anterior to the lambda, 2 mm right lateral to the midline, and 3 mm deep with respect to the surface of the skull. Upon confirming tumors with bioluminescent imaging (BLI, LagoX), mice were randomized to five groups: TNP once a week (TNP1×) or twice a week (TNP2×), MMAE, ferumoxytol, or PBS. The total cumulative doses for each drug regimen were: TNP1× (0.3 mg/Kg of MMAE, 25 mg/Kg of Fe), TNP2× (0.6 mg/Kg of MMAE, 50 mg/Kg of Fe), ferumoxytol (25 mg/Kg), and MMAE (0.3 mg/Kg). All therapeutic agents or PBS were administrated i.v. For combination treatment with radiation therapy, mice were treated with radiation (10 Gy), and then administered TNP 3 h-post-radiation. Prussian Blue staining was performed using an Iron Stain Kit (Sigma) according to the manufacturer's instructions. The tissue slides were deparaffinized and hydrated, and then incubated in working iron stain solution for 10 mins to allow the iron to be bound by the dye. After rinsing with deionized water, the tissue was stained with Nuclear Fast Red solution (Sigma) for 5 mins. The tissue was then dehydrated with alcohol and xylene and mounted for imaging. For immunofluorescent staining, tissues were treated with similar processes and stained with cleaved caspase-3 (Cell Singling) after deparaffinization. All animal maintenance, handling, surveillance, and experimentation were performed in accordance with and under approval from the Stanford University Administrative Panel on Laboratory Animal Care.

### BLI

BLI was performed twice a week after tumor implantation on a Lago (Spectral Instruments Imaging, Tucson) until the end of the study. D‐luciferin firefly potassium salt solution (Biosynth) was prepared at 30 mg/mL and injected intraperitoneally at a dose of 4.5 mg luciferin per mouse before every imaging session. Mice were imaged and total flux values (photons/s) were obtained and analyzed with Aura software.

### MRI

Tumor delivery of TNPs in GBM-bearing mice was assessed using MRI. Our TNPs are composed of ferumoxytol (AMAG Pharmaceuticals), which offers superparamagnetic signal effects on T2-weighted MR images. U87-MG bearing mice underwent MRI before and 24 h after i.v. injection of TNPs or corresponding controls. MRI studies of GBM-bearing mice were performed on a 11.7T MR scanner (Bruker) with a field of view of 30 mm × 30 mm and a slice thickness of 0.5 mm for the following acquisitions : T2-weighted fast spin echo (FSE) repetition time (TR): 3,500 ms, echo time (TE): 54 ms, flip angle α: 180°; multi-slice multi-echo (MSME): TR: 3,000 ms, TE: 7.4-50.5 ms, α: 180°.

### Biodistribution analysis

The biodistribution of TNPs or corresponding controls was investigated using ICP-OES analysis. Briefly, 6- to 8-week-old female NU/J homozygous nude mice (n = 3) were i.v. injected with TNP (0.3 mg/Kg of MMAE, 25 mg/Kg of Fe) once a week (TNP1×) or twice a week (TNP2×), MMAE (0.3 mg/Kg), ferumoxytol (25 mg/Kg), or PBS. At 24 h after the last treatment, mice were euthanized and different organs, including the brain, heart, lung, liver, kidney, and spleen, were harvested. Tissues were weighed and cut in small pieces and treated with nitric acid (3M) and hydrogen peroxide (1M) solution at 100 °C overnight to completely digest samples. The samples were diluted with deionized water, filtered through a 0.44 μm strainer and subjected to ICP-OES analysis to determine ferumoxytol concentration. Standard calibration curves were generated with a solution of ferumoxytol. Sections of formalin-fixed and paraffin-embedded tissue were deparaffinized with xylene, rehydrated, and stained with hematoxylin and eosin (H&E) to observe necrosis. Representative images were captured using a Keyence microscope.

### Statistical analysis

Cell experiments were performed in biological triplicates, in three independent experiments. MRI and BLI images presented are representative of three independent experiments. Results are presented as mean ± SD unless stated otherwise. Tumor relaxation times and BLI signals were compared within multiple experimental groups using a one-way analysis of variance (ANOVA). Results were analyzed using a no matching or pairing ordinary test to compare two or more groups with Tukey's multiple comparison test, with a single pooled variance. Kaplan-Meier survival curves were compared using the log‐rank test. The level of significance was set at *P* < 0.05, as compared with the control group. Statistical analyses were carried out with Prism 9.3.0 software (GraphPad).

## Results

### Novel TNPs can be selectively cleaved by Cat B and release potent toxin MMAE

**Figure 1:**
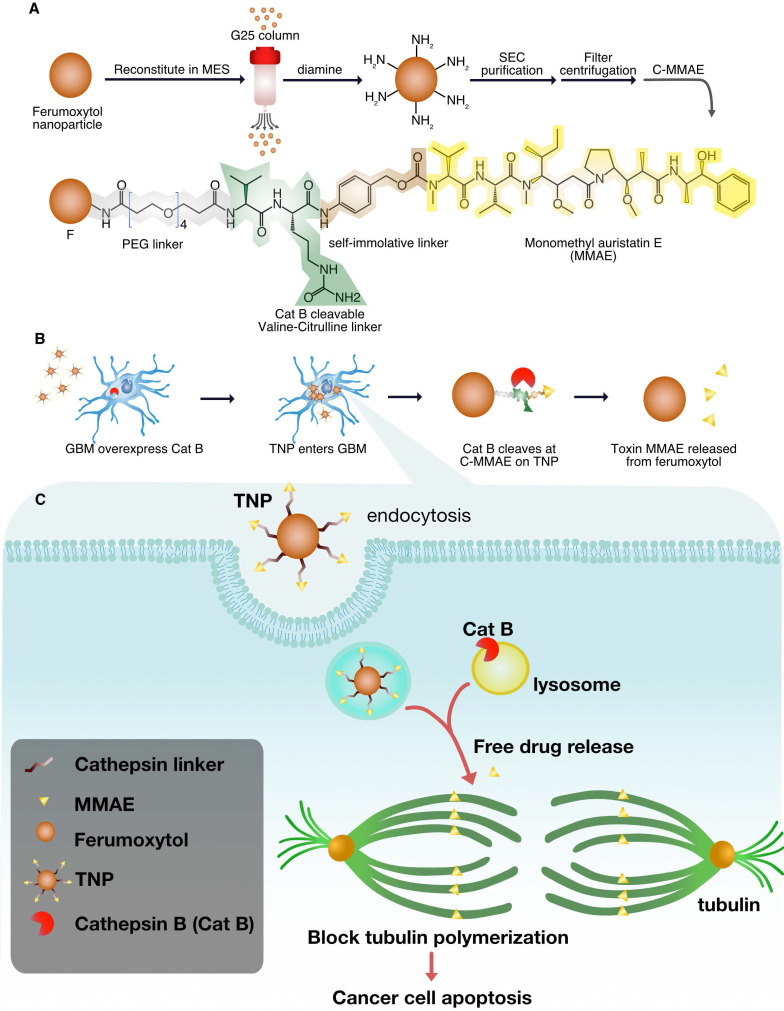
** TNP design.** (A) Synthesis of TNPs. (B) TNPs enter cancer cells and Cat B cleaves drug MMAE off the TNPs. (C) Lysosomal Cat B cleaves TNPs and releases MMAE to inhibit tubulin polymerization and cause tumor cell death.

**Figure 1A** illustrates the concept for Cat B-activatable TNPs. The complex consists of a nanoparticle core and peptide prodrugs. The core is a ferumoxytol iron oxide nanoparticle with an iron oxide crystal coated with polysaccharide dextran. The superparamagnetism of the core allows for MRI of the TNPs. The peptide prodrug includes a potent tubulin inhibitor, MMAE, that has been investigated in many clinical trials for cancer treatment 16. This potent anti-cancer drug is harnessed by a dipeptide valine-citrulline recognition sequence and the whole peptide prodrug is coupled to the ferumoxytol surface through a diamine molecule (see Materials and Methods for details). The TNPs can be specifically cleaved between two peptides by tumor-associated Cat B, releasing free MMAE, inhibiting tubulin polymerization, and inducing cell death (**Figure 1A-B**, **Figure S1**). Thus, the drug is rendered non-toxic until activated, as demonstrated previously for the cathepsin-activated prodrug 6. This new TNP is a ferumoxytol-based cleavable monomethyl auristatin, and we investigate its antitumor activity and imaging capability in GBM.

### Physicochemical properties of TNPs

**Figure 2:**
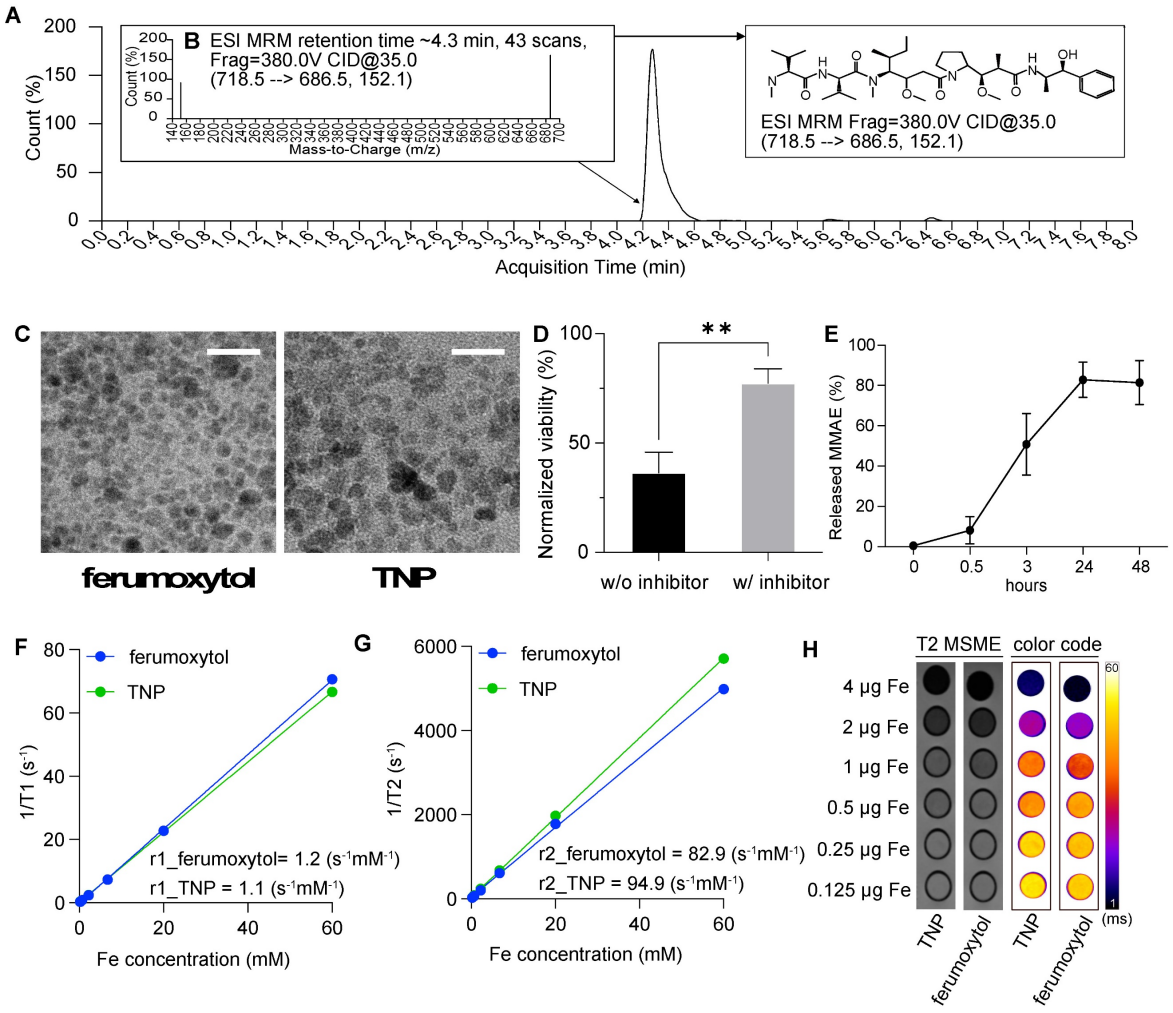
**TNP characterization.** (A) Chromatography of free MMAE released from the TNPs. (B) Product ion of free MMAE. (C) Representative TEM image of the original ferumoxytol (left) and TNPs (right). (D) Activation of TNP is cathepsin-dependent. (E) MMAE releasing profile of TNP. (F) T1-relaxivity and (G) T2-relaxivity were measured from changes in relaxation time at different concentrations of ferumoxytol or TNP. (H) MR images of increasing concentrations of TNP nanoparticles in Ficoll solution on T2-weighted MSME sequences and color-coded T2-maps using the FIRE scheme. Scale bars represent 20 nm.

We characterized the TNPs by liquid chromatography-mass spectrometry-mass spectrometry (LC/MS-MS), TEM, dynamic light scattering (DLS), NMR, and MRI. We first studied TNP activation to ensure that MMAE can be released when peptide prodrugs are conjugated onto ferumoxytol nanoparticles. **Figure 2A-B** show our analysis of co-incubation of TNPs (10 mM) and Cat B enzyme (20 μg/mL). The ratio between nanoparticle and enzyme was chosen based on previous studies and a pilot experiment (**Figure S2**)^17^,^18^. MRM analysis identified released MMAE at its ionized fragments of 152.1 and 686.5 (**Figure 2B**), confirming that our TNPs retain the activatable character of a peptide prodrug upon exposure to Cat B.

Under TEM, the iron oxide nanoparticles maintained the same morphology upon functionalization, as shown in **Figure 2C**. Cat B treatment of TNP cleaved 83% of the drug as was observed by LC/MS-MS assay (**Figure 2E**). Co-incubation of TNP and the cathepsin inhibitor with cancer cells resulted in a higher viability of the cancer cells compared to cells incubated with TNP in the absence of cathepsin inhibitor, indicating that the drug release mechanism of TNP is dependent on cathepsin expression in the GBM cells (**Figure 2D**). Incubating TNP in acidic solution without Cat B showed a low release of MMAE, indicating TNP activation is dependent on the presence of Cat B (**Figure S3**). By measuring the changes in relaxation time at different iron concentrations via NMR, we confirmed that the modified nanoparticles had a similar r1 (1.1 and 1.2 mM^-1^s^-1^ for TNP and ferumoxytol, respectively) and r2 (94.9 and 82.9 mM^-1^s^-1^ for TNP and ferumoxytol, respectively) compared to the original ferumoxytol nanoparticles (**Figure 2D-E**). This result indicates that the peptide prodrug modification on ferumoxytol does not alter the relaxivities of the nanoparticles. Similarly, MR images of the nanoparticle suspensions in a 50% Ficoll solution demonstrated that the conjugated TNPs had similar T2 contrast enhancement as the original ferumoxytol (**Figure 2F**).

Table 1 summarizes our characterization of the TNPs. DLS measurements showed the expected increase in nanoparticle size from 31 ± 1 nm to 41 ± 5 nm after conjugation with peptide prodrug. Amine group addition and drug attachment also changed the charge of the nanoparticle: negative (-17 mV) for ferumoxytol to positive (16 mV) of TNP intermediate and to neutral (5 mV) for TNPs, according to zeta potential measurements.

### TNPs enter lysosomes in GBM cells

**Figure 3:**
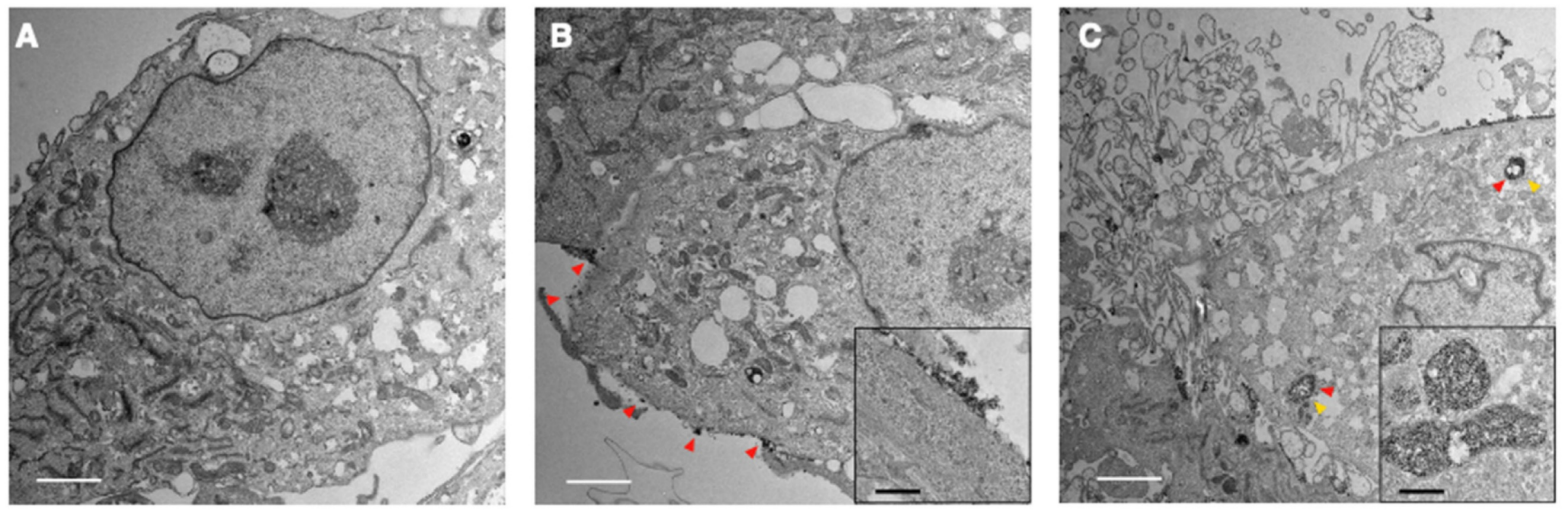
** Representative TEM images of GBM cells exposed to TNPs.** U87-MG cells were incubated with TNPs at a concentration of 4 nM and observed under TEM at (A) 0, (B) 30, and (C) 120 min post-incubation. Secondary lysosomes are indicated by yellow arrowheads. Nanoparticles are indicated by red arrowheads. White scale bars are 2 μm. Inset shows zoomed-in views of nanoparticles around the cell surface or in the lysosomes. Black scale bars in the insets are 400 nm.

To investigate the entry of TNP and its location inside the cells, we incubated TNPs (4 nM) with a human GBM cell line, U87-MG, for TEM observations at different timepoints in comparison with a naïve control (**Figure 3A**). At 30 min post-incubation, TNP largely adhered to the surface of U87-MG cells (**Figure 3B**). Adhered nanoparticles were subsequently taken up by cells with longer incubation. As shown in **Figure 3C**, electron microscopy of treated GBM cells revealed nanoparticle-containing lysosomes/endosomes 120 min post-incubation (red arrowheads indicate nanoparticles). These TEM images demonstrate that our new TNPs can enter GBM tumor cells and reach the lysosome compartments where Cat B enzymes are located. Nejadnik et al used a lysosome tracker and fluorescein-labeled ferumoxytol to show that ferumoxytol is located in lysosomes/endosomes in the cellular compartment. Khurana et al also found that iron oxide nanoparticles mainly accumulate in the secondary lysosomes after entering cells. Our findings are consistent with previous reports that iron oxide nanoparticles including ferumoxytol accumulate in lysosomes after entering cells 19, 20.

### Novel TNPs enable cellular uptake and anti-tumor effects in human GBMs

**Figure 4:**
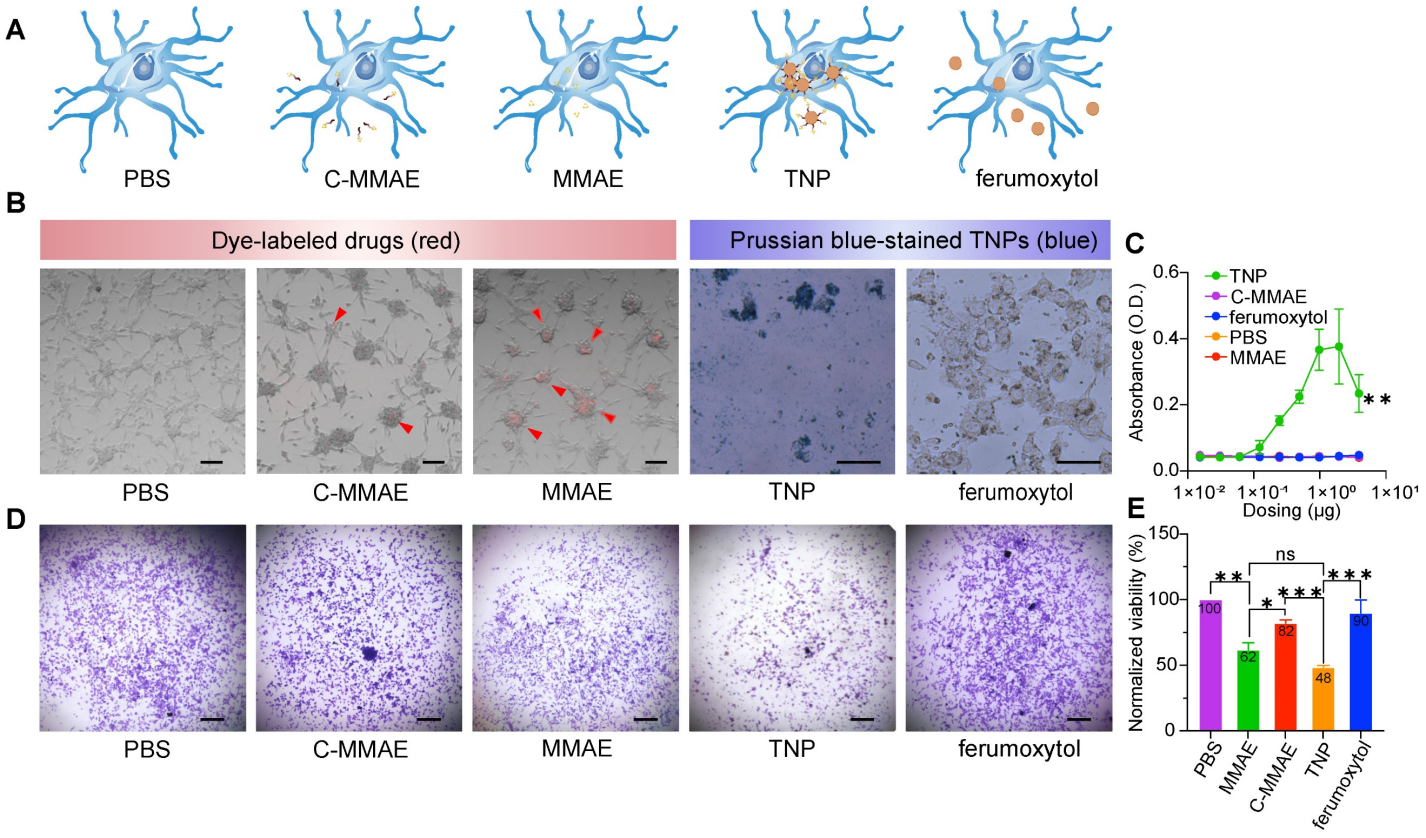
**TNPs are internalized into GBM cells and kill tumor cells.** U87-MG cells were exposed to (A) PBS, peptide prodrug (C-MMAE), free drug (MMAE), TNP and unconjugated ferumoxytol. (B) Internalized drugs or nanoparticles were visualized under a microscope. Internalizations are indicated by red arrowheads. (C) Internalized nanoparticles were measured by UV-Vis. Cell viability was measured by (D) crystal violet staining and (E) the cell titer blue assay. * p < 0.05, ** p < 0.005, one-way ANOVA.

To determine the effects of TNP treatment on GBM cells, we studied the uptake rate and viability of U87-MG cells. We selected the U87-MG human cell line because it is a Cat B-positive GBM cell line 21. We compared five groups: PBS, free drug (MMAE), peptide prodrug (cleavable MMAE, C-MMAE), TNP, and ferumoxytol (**Figure 4A**). Drugs without ferumoxytol nanoparticles (C-MMAE and MMAE) were labeled with Alexa Fluor 555 and nanoparticles (TNP and ferumoxytol) were stained with Prussian blue after incubation for visualization of the cellular uptake, as shown in **Figure 4B**. We also investigated the relationship between nanoparticle uptake and dosing concentration by measuring the UV-Vis absorbance of nanoparticles at wavelength 450 nm 15 at various concentrations (**Figure 4C**). We selected the dose range of TNP based on previous studies, corresponding to the half maximal inhibitory concentration of MMAE in tumor cells 22,23. In cellular uptake assays, U87-MG took up TNP and MMAE after 72 h of incubation (**Figure 4B-C**) and the uptake rate of TNP was concentration dependent (**Figure 4C**). However, in peptide prodrug-treated cells, only a limited amount was taken up by U87-MG cells. The incorporation of peptide reduced the possibility that MMAE was endocytosed by GBM cells. For unconjugated ferumoxytol, the Prussian staining and UV-Vis absorbance results both demonstrated minimal uptake by GBM cells, which is consistent with previous reports that cancer cells have a limited ability to take up the non-modified ferumoxytol nanoparticles 24.

Next, we investigated the effect of TNPs on GBM cell viability. The induction of cellular apoptosis from TNP exposure was observed on confocal microscope images (**Figure S4A-B**, indicated by white arrows) and TEM images (**Figure S4C**). The endocytosed agents containing valine-citrulline peptides can be activated by Cat B in lysosomes to release therapeutic molecules. U87-MG cells were exposed to MMAE, C-MMAE, ferumoxytol, TNP, or PBS for 72 h and stained with crystal violet to visualize the living and adherent cells. Treatment at 1 nM with both MMAE and TNP induced significant loss of cell viability compared to C-MMAE, ferumoxytol, or PBS (**Figure 4D**). Quantitative analysis showed that TNP induced ~52% cell death (48% viable cells) after a 72 h incubation period while MMAE induced ~38% cell death. C-MMAE treatment at the same drug concentration demonstrated ~18% cell death (82% viable cells) while PBS and ferumoxytol did not show any significant cytotoxic effects (**Figure 4D-E**). A low amount of C-MMAE accumulation in GBM cells rescued MMAE toxicity in GBMs, suggesting that it is necessary to enter GBM cells to effectively kill cancer cells. The great amount of uptake of TNP and the high GBM cell death indicated that internalized TNP can successfully release MMAE from the nanoparticles and induce cancer cell death. The drug-release system of the valine-citrulline peptides was not disrupted by the conjugation process. Furthermore, nanoparticle carriers significantly improve peptide prodrug delivery into the cells when compared to peptide prodrug alone (p=0.0008).

### TNPs have anti-tumor effects in orthotopic brain tumors

To establish an orthotopic mouse model for GBM, we injected U87-MG cells intracranially into the right parietal lobes of nude mice. The *in vivo* growth of GBM cells has been previously documented 25. We engineered U87-MG cells to express firefly-luciferase-GFP expressing reporter for *in vivo* tumor detection with bioluminescence imaging and confirmed Cat B expression in the tumor cells prior to the study (**Figure S5**).

**Figure 5:**
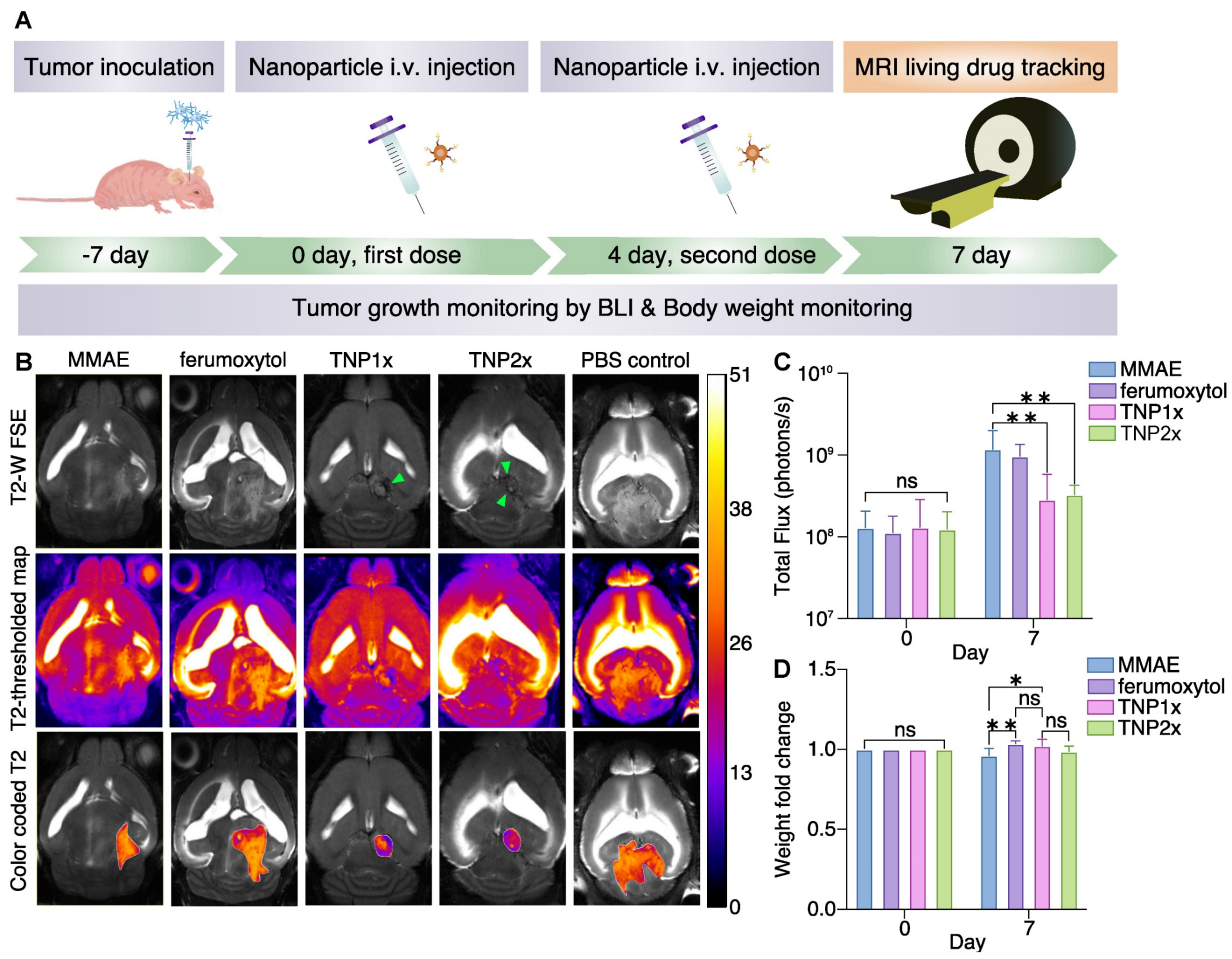
** TNPs inhibit GBM tumor growth.** (A) Treatment scheme of the TNPs. (B) Representative coronal T2-weighted MR images of MMAE-, ferumoxytol-, TNP1×-, TNP2×-, and PBS-injected (control) orthotopic GMB mouse brains (n = 4). Top panel shows FSE images, middle panel shows T2 maps, and bottom panel shows fused images. Green arrows indicate darkened tumors. The regions of interest (ROIs) were defined on the T2-weighted images to identify the tumors. (C) Quantification of the bioluminescent signals in control and treated U87-MG tumors on day 0 and 7. Results are represented as mean ± SEM from three independent experiments. (D) Weight changes of mice on day 0 vs. day 7. Fold change in body weights represents the ratio of body weight after treatment on day 7/body weight before treatment on day 0. TNP1× indicates a total dose of 0.3 mg/Kg and TNP2× indicates a total dose of 0.6 mg/Kg. * p < 0.05, ** p < 0.005, one-way ANOVA.

At 1 to 2 weeks post-intracranial cell injection, tumors were detectable in the brain. We randomized mice into five treatment cohorts of seven mice/group to receive i.v. injection with TNPs once a week (TNP1×) or twice a week (TNP2×), MMAE, ferumoxytol, or PBS. The total cumulative doses for each drug regimen were: TNP1× (0.3 mg/Kg of MMAE, 25 mg/Kg of ferumoxytol), TNP2× (0.6 mg/Kg of MMAE, 50 mg/Kg of ferumoxytol), ferumoxytol (25 mg/Kg), and MMAE (0.3 mg/Kg). We performed a longitudinal time course to study the anti-tumor properties of the TNPs at single or double dosages. The treatment scheme is shown in **Figure 5A**.

To better understand the tumor delivery and retention of TNPs, we subjected U87-MG-bearing mice to MRI studies using a 11.7 T small animal MRI system (Bruker). As shown in **Figure 5B**, both TNP1×- and TNP2×-treated mice showed darkened tumors in the brain (indicated by green arrows), whereas PBS-, MMAE-, and ferumoxytol-treated animals did not demonstrate darkening effects at the tumor areas. The **Figure 5B** also shows shorter T2 relaxation times in the tumors of the treatment groups (TNP1×- and TNP2×) compared to controls. As shown in **Figure 5**, TNP1× and TNP2× treatments were the most effective in slowing down the tumor growth. By contrast, PBS and ferumoxytol-treated tumors showed a significant increase in tumor volume over time. Thus, TNPs could be used for both GBM therapy and noninvasive monitoring of drug delivery and therapy response in orthotopic GBM using MRI. When comparing TNP1× and TNP2×, the more frequent dosing schedule and higher cumulative dosages did not result in smaller tumor sizes, indicating that the extra TNPs may travel to other organs or there is tumor-acquired resistance against the therapeutics. Therefore, a second layer of treatment may be needed to reach the most effective treatment outcome.

In TNP1×- and TNP2×-treated animals, the MR images revealed a smaller tumor size 7 days after the first treatment, but the ferumoxytol-treated mice did not show similar antitumor effects, suggesting that the therapeutic effect is derived directly from the released MMAE entity. The MMAE-treated mice also did not show the same antitumor outcome as the TNP group, which might be due to the limited amount of free drug available to reach the brain tumor. The anti-GBM effects of TNPs are represented by the total flux of BLI signals in **Figure 5C**. As observed, TNP significantly inhibited U87-MG tumor growth after systemic i.v. injection. However, weight loss was observed in MMAE-treated animals, which may be the result of toxicity of the free MMAE to other organs. The TNP1× and TNP2×-treated animals showed only non-significant weight loss (**Figure 5D**), with minimal effects in the TNP2×-treated cohort. Thus, we investigated the biodistribution of TNP1× and TNP2× in nude mice using ICP-OES. The results showed that TNP accumulated in liver and spleen, as expected for nanoparticles, with little accumulation in the brain and heart (**Figure S6**). H&E images showed large necrosis in organs from MMAE-treated animals and minimal necrosis in those from TNP1×-treated animals (**Figure S7**) compared to PBS-treated control group. However, the TNP2×-treated group demonstrated necrosis in kidney parenchyma, which might be due to accumulation of TNP and strong expression of Cat B in kidneys 26. These data indicate the importance of using TNP at a low dose.

### TNPs in combination with radiation therapy impedes tumor growth and improves survival in GBM-bearing animals

**Figure 6:**
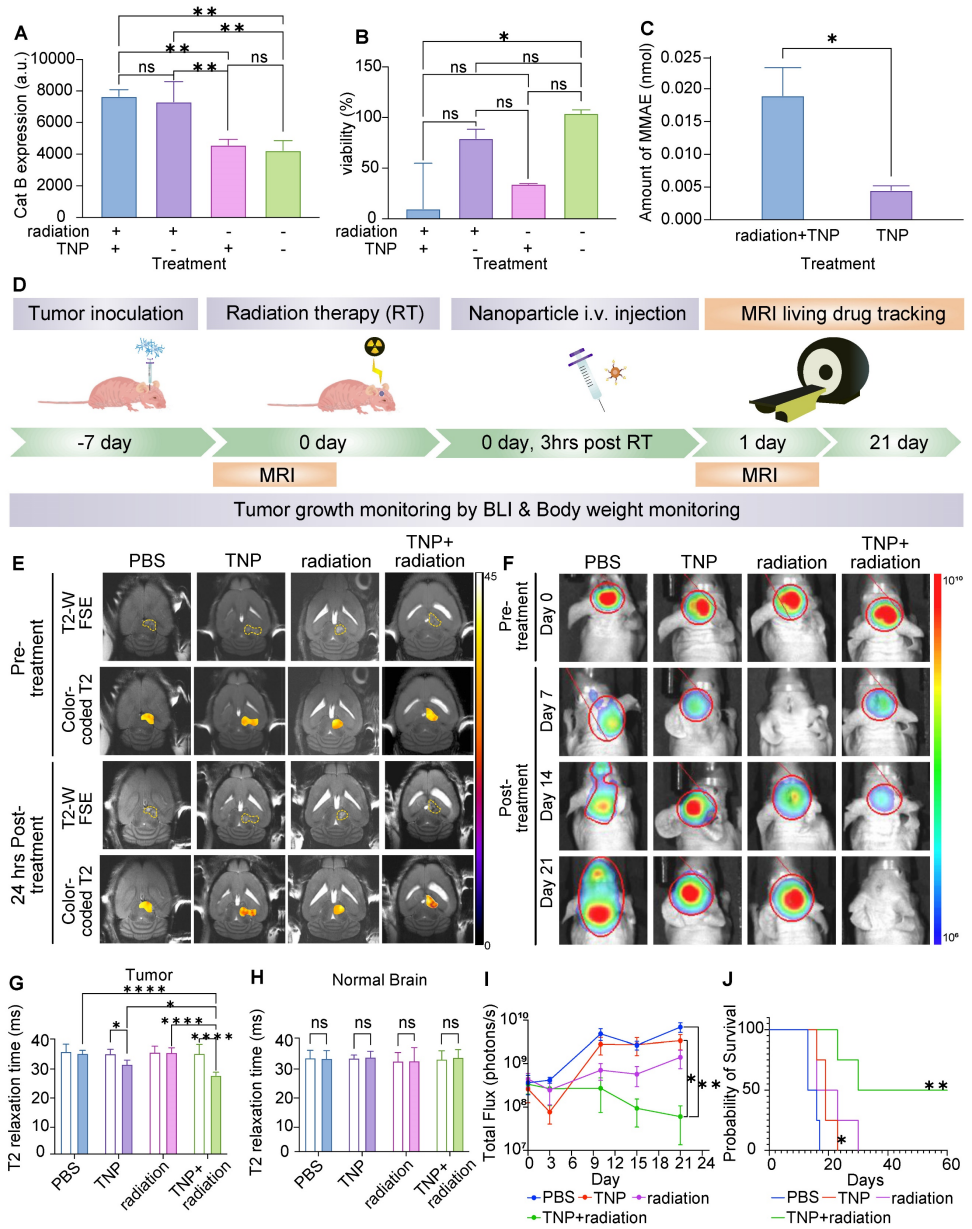
** Combination therapy of TNP and radiation therapy inhibited tumor growth and improved survival.** (A) Cat B expression of U87-MG GBM cells after exposure to TNP and/or radiation therapy (RT). (B) U87 GBM cell viability after receiving TNP and/or RT treatment. (C) The amount of MMAE (M) in culture media of U87-MG cells treated with TNP with or without prior treatment of RT. (D) *In vivo* combination treatment schedule. (E) Representative coronal T2-weighted MR images fused with measured T2 values within the tumor ROIs of TNP-, RT-, TNP+RT- and PBS-injected (control) orthotopic GMB mouse brains. Top two panels show pre-treatment images and corresponding T2 measurements, and bottom two panels show 24 h post-treatment results for comparison. (F) BLI images of animals at day 0, 7, 14, and 21 after first treatment. (G-H) Comparison of T2 relaxation times in tumor and normal brain areas. Empty bars indicate pre-treatment; filled bars indicate post-treatment. (I) Quantification of BLI signals. (J) Kaplan-Meyer survival curves of control and treated mice demonstrate a significant survival benefit of TNP+RT as compared to vehicle, log-rank Mantel-Cox test.

Next, we assessed whether our TNPs could be used in combination with standard clinically approved anti-GBM radiation therapy to benefit the therapeutic outcome. Before proceeding to *in vivo* studies, we screened cells *in vitro* for radiation therapy sensitivity and synergic effects, as shown in **Figure 6A-C**. U87-MG cells were pre-treated with or without radiation therapy, seeded for one day to allow the cells to settle, and then treated with TNP. As shown in **Figure 6A**, we observed an increase in Cat B expression in U87-MG cells after radiation therapy, which was not impacted by subsequent treatment with TNP, regardless of whether cells were pre-treated with radiation therapy or not. Moreover, combining radiation therapy with TNP resulted in the lowest cell viability compared to single treatment or naïve control (**Figure 6B**). When we exposed U87-MG cells to radiation therapy before TNP treatment, we detected a higher amount of free MMAE in the cell supernatant, implying the radiation therapy-treated cells have a greater ability to cleave and release the drug from TNPs.

Human U87-MG cells were responsive to radiation therapy and the combination of radiation therapy and TNP demonstrated the strongest anti-GBM effect (**Figure 6B-C**). Therefore, for our *in vivo* studies we proceeded with U87-MG cells to test the anti-tumor effects of radiation therapy and TNP in combination. At 1-2 weeks post-intracranial injection, we randomized mice into treatment groups based on tumor size. We administered radiation therapy at 10 Gy either alone or in combination with a single dose of TNP (25 mg/Kg, i.v.). The treatment schedule is shown in **Figure 6D**. We injected TNP at 3 h post-radiation therapy, which is a previously optimized schedule 25. We performed a coronal MRI scan to observe the nanoparticle accumulation in the brain (**Figure 6E**) and applied tumor bioluminescence to evaluate the tumor response (**Figure 6F**). The ferumoxytol core enables *in vivo* drug tracking with MRI. Both preclinical and clinical studies also showed the ferumoxytol can be imaged in glioblastoma. Mohanty et al demonstrated that ferumoxytol-based theranostic nanoparticles can reach GBM in a murine model and be imaged with MRI 13. Nasseri and Iv et al reported that intravenously administered ferumoxytol accumulated in glioblastomas in patients, as confirmed with MRI and histology 27,28. Therefore, we overlaid the measured T2 values in the tumors to assess the accumulation of our TNP (**Figure 6E**). When comparing pre-and post-injection images in each group, we found that tumor T2 relaxation times decreased after TNP injection whereas tumor T2 relaxation times did not change before or after treatment with single radiation therapy or PBS. This indicate our TNP can successfully accumulate at tumor and provide imaging ability for tracking after administration. The quantified T2 values on the tumors and normal brains were shown in **Figure 6G-H**. The combination of radiation therapy and TNP showed an enhanced T2 shortening compared to TNP alone, indicating that radiation therapy improved the accumulation of nanoparticles in the brain tumor. T2 relaxation times of normal brain regions were not significantly different between mice treated with TNP alone, radiation therapy alone, radiation therapy with TNP, or PBS, suggesting that TNP did not accumulate in the normal brain after the treatments (**Figure 6H**). Due to the enhanced permeability and retention effect of nanoparticles, it is possible that tumor retention of TNP is improved after receiving radiation therapy. We harvested and stained tumors with Prussian Blue to investigate the accumulation of nanoparticles. Compared to the control group, we found higher iron content in TNP- and TNP+radiation-treated tumors (**Figure S8**), indicating consistent delivery of nanoparticles to the tumor in both cases. The tumors were stained with cleaved caspase-3 to detect apoptotic cells. GBM treated with TNP or combination of TNP+radiation demonstrated higher apoptosis levels compared to PBS-treated controls (**Figure S9**). When comparing apoptosis level between tumor and normal brain areas, images showed multiple apoptotic cells in GBM while no or sporadic apoptotic cells were found in normal brain (**Figure S10**), indicating our TNPs targeted the tumor specifically.

The combination of radiation therapy with TNP strongly inhibited tumor growth as compared to the single drug regimens (**Figure 6F&I**). Radiation therapy alone significantly improved animal survival times in the U87-MG model, and the addition of TNP further augmented the survival benefits of radiation therapy (**Figure 6J**). Both radiation therapy and TNP slowed tumor growth relative to that of vehicle-treated mice (**Figure 6I**). However, only the combination treatment cohort showed reduced tumor growth after 3 weeks of treatment (**Figure 6I**). Further, the combination of TNP and radiation therapy significantly improved survival outcomes when compared to PBS-treated mice (**Figure 6J**). Together, our *in vitro* and *in vivo* data demonstrate great potential of our TNPs to be used in combination with radiation therapy to improve the therapeutic effects and to enable *in vivo* drug tracking.

## Discussion

Our results showed that Cat-B-activatable TNPs, which possess covalently-bond peptide prodrugs, can cause significant cell death in GBM. The TNPs synergized with radiation therapy, leading to GBM regression and significantly improved survival outcomes *in vivo*, thereby revealing a novel therapeutic combinatorial regimen for targeting GBMs. Current GBM therapies include surgical resection followed by radiation therapy with concurrent chemotherapy. However, approximately 90% of GBM patients undergo tumor recurrence, which is partly attributed to distinct inadequacies in chemotherapeutic strategies 29-31. Nearly all large molecules and the majority of small molecular drugs, including tyrosine kinase inhibitors, are effluxed at the blood brain barrier by the membrane-bound transport proteins P-gp and Bcrp, on the luminal side of endothelial cells and never reach therapeutic levels in the brain 29. Another challenge in finding effective treatments involves the multiple molecular pathways of GBMs. Identifying only one target is insufficient, as this method does not account for the considerable array of genetic and epigenetic heterogeneities found within and among GBM patients 1. Our current TNP approach addresses most of the above challenges. We built our TNPs on a nanocarrier platform that facilitates drug delivery and accumulation by an enhanced permeability and retention (EPR) effect. Preclinical and clinical studies showed that ferumoxytol nanoparticles accumulate in glioblastomas. Mohanty et al demonstrated that ferumoxytol-based theranostics accumulate in GBM in a murine model and can be imaged with MRI 13. Nasseri and Iv et al have reported that ferumoxytol nanoparticles accumulate in GBM in patients 27,28. Indeed, the delivery of our TNPs to the tumor was superior, as shown by MRI, leading to improved antitumor effects in comparison to free-drug-treated cohorts. Once in the tumor, our TNPs undergo activation by the enzyme Cat B, which is overexpressed by tumor cells, and demonstrate specific tumor killing with minimal systemic toxicity. We designed our TNPs to cause tumor cell death and disrupt the tumor microenvironment rather than focusing on a specific GBM target. The diffusible drug MMAE released from dead cells had a sufficiently high concentration to kill neighboring tumor cells either directly or indirectly by altering the tumor microenvironment, making this a highly effective therapeutic strategy 32,33. Additionally, the use of iron-oxide nanoparticles enables real-time monitoring of drug delivery and therapy response. This approach can also be further extended to other GBM therapies to facilitate personalized medicine for GBM patients. Our TNPs combined with radiation therapy showed marked antitumor effects in Cat-B-expressing GBMs when compared with standard radiation therapy, suggesting that our TNP approach has marked potential for clinical translation.

Studies by Agemy *et al.* showed that theranostics with the tumor-vasculature-targeting peptide CGKRK achieved exceptional efficacy in eliminating a lentivirus-induced GBM model; however, this treatment failed to completely inhibit GBM progression induced by orthotopic inoculation of a GBM cell line 34. In contrast, our TNPs significantly inhibited aggressive U87-MG GBMs and achieved almost complete remission of orthotopic U87-MG GBM tumors when combined with radiation therapy. Recent studies have confirmed that Cat B is rarely expressed in normal brain tissue but is overexpressed in GBM and GBM stem cells (GSCs) 35,36. This phenomenon gives our TNPs the opportunity to eliminate not only GBM cells but also GSCs to reduce disease recurrence 37. In GBM, Cat B is primarily located in the invasive margins of tumor infiltration and neovascularization 38. Cat B expression is positively correlated with disease progression, and in high-grade gliomas, Cat B exhibits significant levels of expression 39. A study using gadolinium-based nanoparticles with Cat B-responsive linker has successfully proved the delivery of chemotherapeutic drug in the tumor 40. Therefore, our TNP approach could be further investigated for the treatment of recurrent high-grade GBMs and their impact on deconstructing neovascularization for increasing drug accumulation. Further, because tumor Cat B activates the TNPs to release the potent anticancer drug MMAE specifically in tumor tissue, the risk of cardiotoxicity observed in other vascular disrupting agent studies is minimized 41. Hence, our TNP approach holds promise for clinical trials compared with other vascular disrupting agents.

It is known that MMAE can sensitize tumors to irradiation only with sufficient time and concentration by enhancing ionizing radiation-induced DNA double-strand breaks 42,43. Lisa *et al.* reported a significant increase in γH2AX foci formation and CHK1 activation in irradiated cancer cells treated with free drug MMAE 43. Here, we demonstrated synergistic *in vitro* and *in vivo* therapeutic effects of our TNPs on irradiated GBM cells. *In vivo,* our TNPs can create a local high concentration of drug at the brain tumor, which offers potential for synergy with irradiation. MRI demonstrated more T2 signal drops at the brain tumor in the TNP+ radiation therapy-treated group compared with the TNP-treated group, which is consistent with the literature finding that radiation can increase nanoparticle accumulation in brain tumors 44. Furthermore, when combined with radiation therapy, our TNPs significantly reduced GBM growth and had a synergistic effect on survival outcomes in GBM-bearing mice. This could be attributed to the TNP effects of arresting GBM cells in the G2-M phase and enhancing clonogenic cell death, which occur when TNPs are combined with irradiation, as well as the increase in tumor-associated proteases (Cat B), which can activate more TNPs to release the drug. More comprehensive studies, including investigations of the synergy between irradiation and protease-cleaving activity, are required to elucidate the underlying mechanisms.

In a previous study, chlorotoxin nanoparticles coupled with miR-21 oligonucleotides enhanced apoptosis and, to a lesser extent, improved animal survival in GBM-bearing mice when combined with sunitinib-based anti-angiogenic therapies 45. Jing *et al.* demonstrated CD133-targeted near-infrared photoimmunotherapy (PIT) for CD133 GSCs in both subcutaneous and invasively growing brain tumors. However, these effects were accompanied by severe cerebral edema a few hours after treatment, which was probably associated with PIT (66). In contrast, the activation mechanism of our TNPs is built-in, i.e., the TNPs are activated by the tumor itself and do not rely on external stimuli such as near-infrared light, radiofrequency ablation, or thermal induction. We have also demonstrated the possibility of non-invasively imaging TNP delivery and therapy response in brain tumors by MRI using ferumoxytol, which has been approved by the FDA.

Further studies are warranted to improve the therapeutic efficacy of our TNPs. For example, because our TNPs are iron-labeled, we could potentially combine MRI with high-intensity focused ultrasound to achieve additional MRI-guided tumor ablation. The TNPs extravasate across organs of the reticuloendothelial system (RES) containing microvessels 46 with sinusoids; therefore, there is a need for strategies to enhance tumor-selective delivery and reduce the potential for toxic side effects in the RES. Further, our MRI technique does not visualize TNP activation; thus, as future work, we could further refine the TNPs to enable real-time imaging of the tumor-selective drug release. Currently, differences in relaxivities between non-activated and activated products are too small to be visualized by MRI *in vivo*. To facilitate monitoring of drug release kinetics, future generations of TNPs could produce MRI contrast either during drug activation or as a consequence of the drug's pharmacological activity. In summary, our TNPs provide improved and selective tumor therapy, with reduced or eliminated toxic side effects. Thus, these TNPs hold significant clinical potential for improving both targeted therapy and diagnostic capacity in GBM patients.

## Conclusion

We have demonstrated that our Cat-B-responsive TNPs are successfully taken up by GBM cells and can induce cancer cell death. The FDA-approved ferumoxytol core in the TNPs enables us to track the location of our therapeutic agents using MRI. These novel TNPs offer *in vivo* monitoring of drug location, enhanced tumor cell internalization, and effective control of tumor growth with radiation therapy, the current clinical standard treatment. Therefore, these novel TNPs hold great potential for improving survival in GBM.

## Supplementary Material

Supplementary figures.

## Figures and Tables

**Table 1:** Summary of TNP characterization.

Samples	Hydrodynamic size (nm)	Polydispersity index	Zeta Potential (mV)
**Ferumoxytol**	31 ± 1	0.27 ± 0.09	-17 ± 5
**TNP intermediate**	36 ± 5	0.29 ± 0.10	16 ± 3
**TNP**	41 ± 5	0.32 ± 0.11	6 ± 3

## Abbreviations

GBM: glioblastoma
TNP: theranostic nanoparticle
Cat B: cathepsin B
MMAE: monomethyl auristatin E
MRI: magnetic resonance imaging
ADC: antibody-drug conjugate
PSMA: prostate-specific membrane antigen
FDA: Food and Drug Administration
i.v.: intravenous
HOBt: hydroxybenzotriazole
EDC: 1-ethyl-3-(3-dimethylaminopropyl)carbodiimide
C-MMAE: cathepsin-cleavable monomethyl auristatin E
PBS: phosphate buffered saline
TEM: transmission electron microscope
ICP-OES: inductively coupled plasma optical emission spectrometer
MRM: multiple reaction monitoring
D_2_O: deuterium oxide
NMR: nuclear magnetic resonance
T1: Relaxation time 1
T2: relaxation time 2
r1: relaxivity 1
r2: relaxivity 2
GFP: green fluorescence protein
BLI: bioluminescent imaging
FSE: fast spin echo
TR: repetition time
TE: echo time
MSME: multi-slice multi-echo
H&E: hematoxylin and eosin
DLS: dynamic light scattering
GSCs: glioblastoma stem cells
EPR: enhanced permeability and retention
PIT: photoimmunotherapy
RES: reticuloendothelial system

## References

[1] Prados MD, Byron SA, Tran NL, Phillips JJ, Molinaro AM, Ligon KL (2015). Toward precision medicine in glioblastoma: the promise and the challenges. Neuro Oncol.

[2] Jue TR, McDonald KL (2016). The challenges associated with molecular targeted therapies for glioblastoma. J Neurooncol.

[3] Nguyen LT, Touch S, Nehme-Schuster H, Antoni D, Eav S, Clavier J-B (2013). Outcomes in newly diagnosed elderly glioblastoma patients after concomitant temozolomide administration and hypofractionated radiotherapy. Cancers (Basel).

[4] Nieder C, Andratschke N, Wiedenmann N, Busch R, Grosu AL, Molls M (2004). Radiotherapy for high-grade gliomas. Does altered fractionation improve the outcome?. Strahlenther Onkol.

[5] Luo D, Wang X, Walker E, Springer S, Ramamurthy G, Burda C (2022). Targeted Chemoradiotherapy of Prostate Cancer Using Gold Nanoclusters with Protease Activatable Monomethyl Auristatin E. ACS Appl Mater Interfaces.

[6] Gan HK, van den Bent M, Lassman AB, Reardon DA, Scott AM (2017). Antibody-drug conjugates in glioblastoma therapy: the right drugs to the right cells. Nat Rev Clin Oncol.

[7] Qi R, Wang Y, Bruno PM, Xiao H, Yu Y, Li T (2017). Nanoparticle conjugates of a highly potent toxin enhance safety and circumvent platinum resistance in ovarian cancer. Nat Commun.

[8] Holzgreve A, Biczok A, Ruf VC, Liesche-Starnecker F, Steiger K, Kirchner MA (2021). PSMA Expression in Glioblastoma as a Basis for Theranostic Approaches: A Retrospective, Correlational Panel Study Including Immunohistochemistry, Clinical Parameters and PET Imaging. Front Oncol.

[9] Zhu Z, Du S, Du Y, Ren J, Ying G, Yan Z (2018). Glutathione reductase mediates drug resistance in glioblastoma cells by regulating redox homeostasis. J Neurochem.

[10] Rempel SA, Rosenblum ML, Mikkelsen T, Yan P-S, Ellis KD, Golembieski WA (1994). Cathepsin B Expression and Localization in Glioma Progression and Invasion. Cancer Res.

[11] Koh SP, Wickremesekera AC, Brasch HD, Marsh R, Tan ST, Itinteang T (2017). Expression of Cathepsins B, D, and G in Isocitrate Dehydrogenase-Wildtype Glioblastoma. Front Surg.

[12] Gondi CS, Rao JS (2013). Cathepsin B as a cancer target. Expert Opin Ther Targets.

[13] Mohanty S, Chen Z, Li K, Morais GR, Klockow J, Yerneni K (2017). A Novel Theranostic Strategy for *MMP-14* -Expressing Glioblastomas Impacts Survival. Mol Cancer Ther.

[14] Aghighi M, Golovko D, Ansari C, Marina NM, Pisani L, Kurlander L (2015). Imaging Tumor Necrosis with Ferumoxytol. PLoS One.

[15] Yuan H, Wilks MQ, Normandin MD, El Fakhri G, Kaittanis C, Josephson L (2018). Heat-induced radiolabeling and fluorescence labeling of Feraheme nanoparticles for PET/SPECT imaging and flow cytometry. Nat Protoc.

[16] Li C, Zhang C, Li Z, Samineni D, Lu D, Wang B (2020). Clinical pharmacology of vc-MMAE antibody-drug conjugates in cancer patients: learning from eight first-in-human Phase 1 studies. MAbs.

[17] Ansari C, Tikhomirov GA, Hong SH, Falconer RA, Loadman PM, Gill JH (2014). Development of Novel Tumor-Targeted Theranostic Nanoparticles Activated by Membrane-Type Matrix Metalloproteinases for Combined Cancer Magnetic Resonance Imaging and Therapy. Small.

[18] Ma Y, Dela Cruz-Chuh J, Khojasteh SC, Dragovich PS, Pillow TH, Zhang D (2019). Carfilzomib Is Not an Appropriate Payload of Antibody-Drug Conjugates Due to Rapid Inactivation by Lysosomal Enzymes. Drug Metab Dispos.

[19] Nejadnik H, Taghavi-Garmestani S-M, Madsen SJ, Li K, Zanganeh S, Yang P (2018). The Protein Corona around Nanoparticles Facilitates Stem Cell Labeling for Clinical MR Imaging. Radiology.

[20] Khurana A, Chapelin F, Beck G, Lenkov OD, Donig J, Nejadnik H (2013). Iron administration before stem cell harvest enables MR imaging tracking after transplantation. Radiology.

[21] Zhang X, Wang X, Xu S, Li X, Ma X (2018). Cathepsin B contributes to radioresistance by enhancing homologous recombination in glioblastoma. Biomed Pharmacother.

[22] Raposo Moreira Dias A, Bodero L, Martins A, Arosio D, Gazzola S, Belvisi L (2019). Synthesis and Biological Evaluation of RGD and isoDGR-Monomethyl Auristatin Conjugates Targeting Integrin α(V) β(3). ChemMedChem.

[23] Ekholm F, Ruokonen S-K, Redón M, Pitkänen V, Vilkman A, Saarinen J (2018). Hydrophilic Monomethyl Auristatin E Derivatives as Novel Candidates for the Design of Antibody-Drug Conjugates. Separations.

[24] Lazaro-Carrillo A, Filice M, Guillén MJ, Amaro R, Viñambres M, Tabero A (2020). Tailor-made PEG coated iron oxide nanoparticles as contrast agents for long lasting magnetic resonance molecular imaging of solid cancers. Mater Sci Eng C.

[25] Wu W, Klockow JL, Mohanty S, Ku KS, Aghighi M, Melemenidis S (2019). Theranostic nanoparticles enhance the response of glioblastomas to radiation. Nanotheranostics.

[26] Saudenova M, Promnitz J, Ohrenschall G, Himmerkus N, Böttner M, Kunke M (2022). Behind every smile there's teeth: Cathepsin B's function in health and disease with a kidney view. Biochim Biophys Acta Mol Cell Res.

[27] Nasseri M, Gahramanov S, Netto JP, Fu R, Muldoon LL, Varallyay C (2014). Evaluation of pseudoprogression in patients with glioblastoma multiforme using dynamic magnetic resonance imaging with ferumoxytol calls RANO criteria into question. Neuro Oncol.

[28] Iv M, Samghabadi P, Holdsworth S, Gentles A, Rezaii P, Harsh G (2019). Quantification of Macrophages in High-Grade Gliomas by Using Ferumoxytol-enhanced MRI: A Pilot Study. Radiology.

[29] Singh SK, Hawkins C, Clarke ID, Squire JA, Bayani J, Hide T (2004). Identification of human brain tumour initiating cells. Nature.

[30] Minniti G, Niyazi M, Alongi F, Navarria P, Belka C (2021). Current status and recent advances in reirradiation of glioblastoma. Radiat Oncol.

[31] Chen J, Li Y, Yu T-S, McKay RM, Burns DK, Kernie SG (2012). A restricted cell population propagates glioblastoma growth after chemotherapy. Nature.

[32] Fu Z, Li S, Han S, Shi C, Zhang Y (2022). Antibody drug conjugate: the “biological missile” for targeted cancer therapy. Signal Transduct Target Ther.

[33] Shen Y, Yang T, Cao X, Zhang Y, Zhao L, Li H (2019). Conjugation of DM1 to anti-CD30 antibody has potential antitumor activity in CD30-positive hematological malignancies with lower systemic toxicity. MAbs.

[34] Agemy L, Friedmann-Morvinski D, Kotamraju VR, Roth L, Sugahara KN, Girard OM (2011). Targeted nanoparticle enhanced proapoptotic peptide as potential therapy for glioblastoma. Proc Natl Acad Sci U S A.

[35] Bischof J, Westhoff M-A, Wagner JE, Halatsch M-E, Trentmann S, Knippschild U (2017). Cancer stem cells: The potential role of autophagy, proteolysis, and cathepsins in glioblastoma stem cells. Tumour Biol.

[36] Tan DC, Roth IM, Wickremesekera AC, Davis PF, Kaye AH, Mantamadiotis T (2019). Therapeutic targeting of cancer stem cells in human glioblastoma by manipulating the renin-angiotensin system. Cells.

[37] Schonberg DL, Lubelski D, Miller TE, Rich JN (2014). Brain tumor stem cells: molecular characteristics and their impact on therapy. Mol Aspects Med.

[38] Kast RE (2010). Glioblastoma invasion, cathepsin B, and the potential for both to be inhibited by auranofin, an old anti-rheumatoid arthritis drug. Cent Eur Neurosurg.

[39] Ma K, Chen X, Liu W, Chen S, Yang C, Yang J (2022). CTSB is a negative prognostic biomarker and therapeutic target associated with immune cells infiltration and immunosuppression in gliomas. Sci Rep.

[40] Cai H, Xiang Y, Zeng Y, Li Z, Zheng X, Luo Q (2021). Cathepsin B-responsive and gadolinium-labeled branched glycopolymer-PTX conjugate-derived nanotheranostics for cancer treatment. Acta Pharm Sin B.

[41] Gill JH, Loadman PM, Shnyder SD, Cooper P, Atkinson JM, Ribeiro Morais G (2014). Tumor-targeted prodrug ICT2588 demonstrates therapeutic activity against solid tumors and reduced potential for cardiovascular toxicity. Mol Pharm.

[42] Hingorani DV, Allevato MM, Camargo MF, Lesperance J, Quraishi MA, Aguilera J (2022). Monomethyl auristatin antibody and peptide drug conjugates for trimodal cancer chemo-radio-immunotherapy. Nat Commun.

[43] Buckel L, Savariar EN, Crisp JL, Jones KA, Hicks AM, Scanderbeg DJ (2015). Tumor radiosensitization by monomethyl auristatin E: mechanism of action and targeted delivery. Cancer Res.

[44] Joh DY, Sun L, Stangl M, Al Zaki A, Murty S, Santoiemma PP (2013). Selective targeting of brain tumors with gold nanoparticle-induced radiosensitization. PloS One.

[45] Sundstrom JB, Mao H, Santoianni R, Villinger F, Little DM, Huynh TT (2004). Magnetic resonance imaging of activated proliferating rhesus macaque T cells labeled with superparamagnetic monocrystalline iron oxide nanoparticles. J Acquir Immune Defic Syndr.

[46] Cho K, Wang X, Nie S, Chen ZG, Shin DM (2008). Therapeutic nanoparticles for drug delivery in cancer. Clin Cancer Res.

