# Supplementary material for: Tumor protease-activated theranostic nanoparticles for MRI-guided glioblastoma therapy
Source: Theranostics. 2023 Mar 13;13(6):1745–58. doi: 10.7150/thno.79342 (PMC10091873; doi:10.7150/thno.79342)
Supplement: Supplementary file 1 — Supplementary figures. [file thnov13p1745s1.pdf]

Supplement Materials

Tumor protease-activated theranostic nanoparticles for MRI-guided glioblastoma therapy

Ching-Hsin Huang[1]*, Edwin Chang[1,2], Li Zheng[3], Joe Gerald Jesu Raj[1], Wei Wu[1], Laura J. Pisani[1,4], and Heike E. Daldrup-Link[1]

[1] Department of Radiology, Molecular Imaging Program at Stanford (MIPS), Stanford University, CA, U.S.A.

[2] Stanford Center for Innovation in In Vivo Imaging (SCi[3]), Canary Center for Cancer Early Detection, Stanford University, CA, U.S.A.

[3] Sarafan Chemistry, Engineering & Medicine for Human Health (Chem-H), Stanford University, Stanford, CA, U.S.A.

[4] Stanford Center for Innovation in In Vivo Imaging (SCi[3]), James H. Clark Center, Stanford University, CA, U.S.A.

***Corresponding author**:

Ching-Hsin Huang. Ph.D.

265 Campus Drive

School of Medicine, room G2045, Lokey Stem Cell Research Building,

Stanford, CA 94305-5614

chh110@stanford.edu

alternative email: hlaura1017@gmail.com

The nano-theranostic construct is illustrated in **Figure S1** and is composed of three components. The first component is an iron oxide nanoparticle, ferumoxytol, which provides MR imaging ability. The second component is a protease-sensitive dipeptide valine-citrulline, which can be cleaved by cathepsin B at the amide bond between citrulline and the attached therapeutic drug. The third component is the therapeutic anti-mitotic drug MMAE. A self-immolative spacer, para-amino benzyloxycarbonyl, spontaneously undergoes a 1,6-elimination reaction upon cleavage by cathepsin B. This reaction releases p-iminoquinone methide and carbon dioxide, and results in the release of the MMAE. The purpose of this construct is to allow for the controlled release of MMAE in the presence of cathepsin B, which is present in high levels in certain types of cancer cells. The construct is not therapeutically active outside of the tumor microenvironment, thereby limiting of target toxicity.

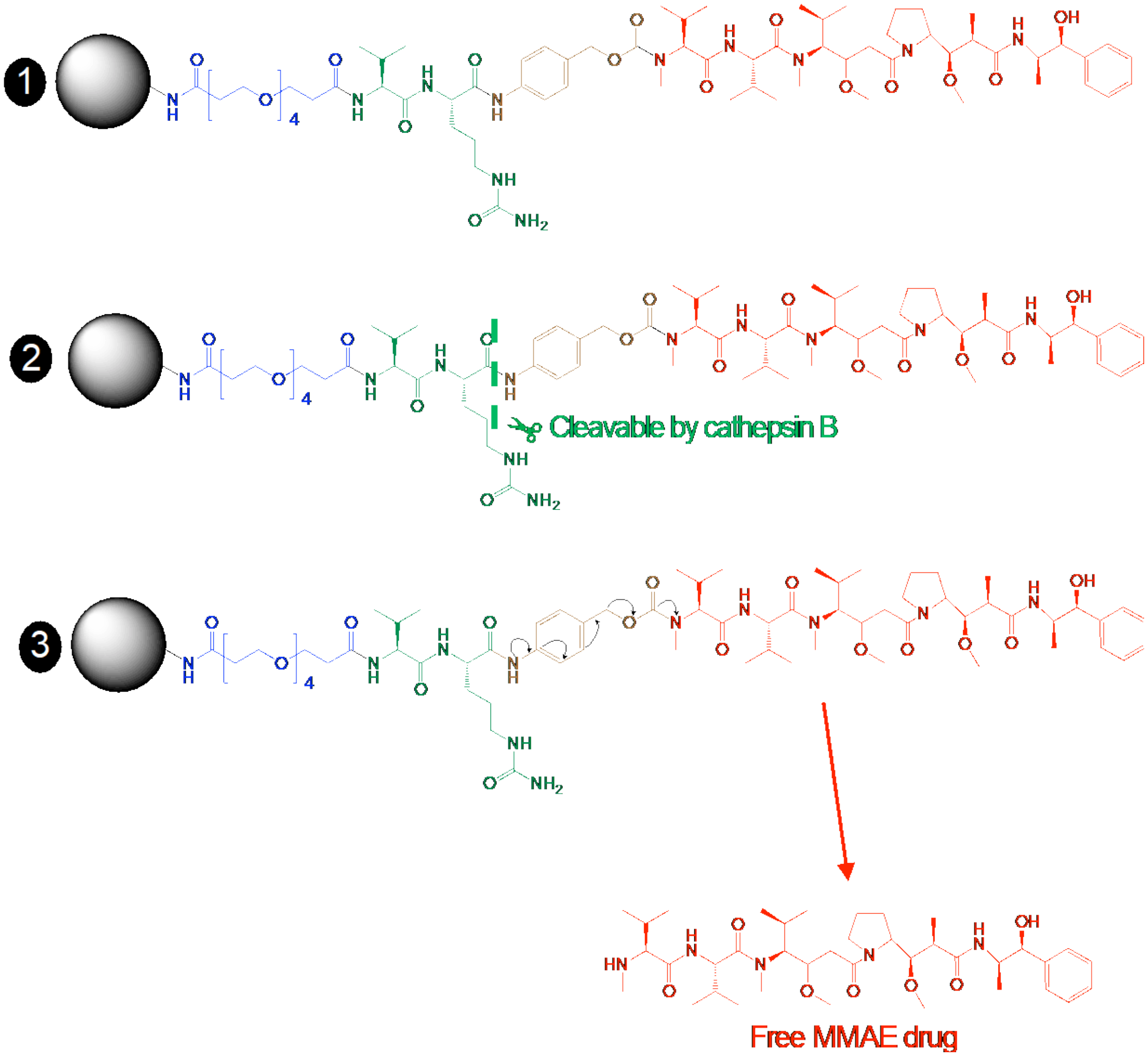

Figure S1. Drug release mechanism of TNP. The TNP is composed of three components: The first component (dark grey) is an iron oxide nanoparticle, ferumoxytol, which provides MR imaging ability. The second component (blue, green and brown) is a protease-sensitive dipeptide valine-citrulline, which can be cleaved by cathepsin B. The third component (red) is the therapeutic anti-mitotic drug MMAE. The protein linker (second component) consists of a valine-citrulline (green) protease recognition sequence and a para-amino benzyl alcohol (self-immolative moiety, in brown). When TNP encounters cathepsin B, it cleaves at the valine-citrulline site and releases the MMAE drug (red).

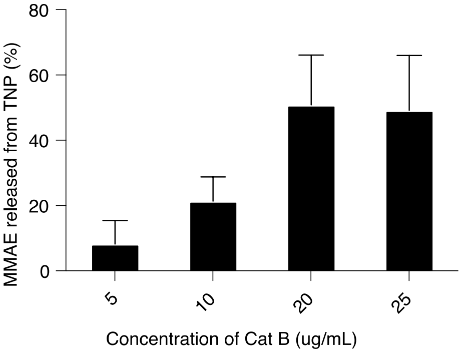

Figure S2. MMAE release from TNP incubated with different concentrations of the Cat B enzyme. 10 mM of TNP was incubated with Cat B at 5, 10, 20, 25 µg/mL. MMAE release was measured by LC/MS-MS.

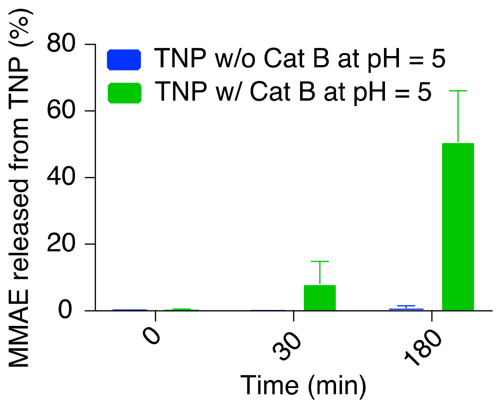

Figure S3. Cat B is required to activate TNP. TNP was suspended in water solution at pH=5.0 at 10 mM concentration with or without the presence of Cat B and incubated at 37 °C. Percentage of released MMAE were measured at 0, 30, and 180 minutes after incubation. Error bars represent Standard Deviation.

The induction of cellular apoptosis from TNP exposure was observed on confocal microscope images (**Figure S4A-B**, indicated by white arrows) and TEM images (**Figure S4C**).

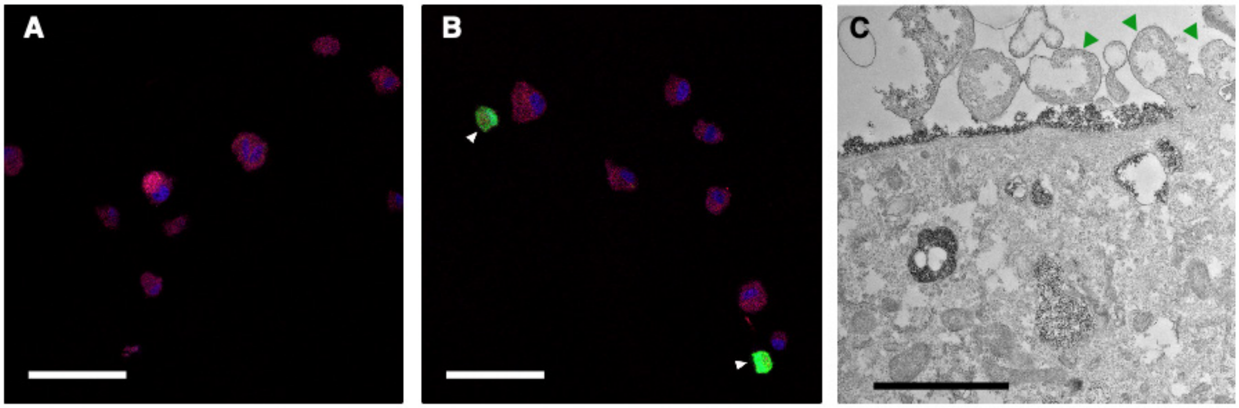

Figure S4. Representative images depicting the effect of apoptosis induction on GBM cells. Confocal images of (A) Control or (B) TNP-treated U87-MG cells were harvested and stained with cleaved-caspase-3. Images show TNP induced apoptosis in GBM cells. White arrows (cells in green) indicate apoptosis. Cells were masked in red and nuclei were stained in blue. (C) TEM image of U87-GBM cells after exposing to TNP. Green arrows indicate the membrane blebbing which is shown in apoptotic cells. Scale bars in (A) and (B) indicate 50 μm. Scale bar in (C) indicate 2 μm

The U87-MG cell line is a Cat B-positive glioblastoma cell line. U87-MG cells and extracted normal brain cells were stained with Cat B. A secondary antibody only sample was used as a negative control. The intracellular Cat B expression in the U87-MG cell line was verified before intracranially implanting tumor cells into animals.

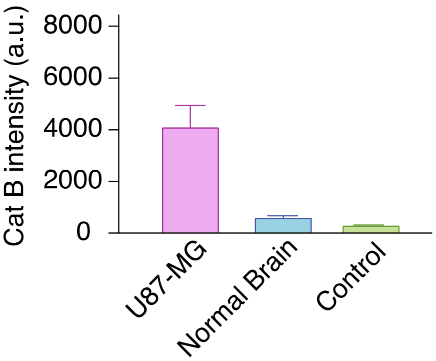

Figure S5. U87-MG cell line is a Cat B-positive glioblastoma cell line. U87-MG cells or normal brain cells were stained with Cat B. Control group represents cells stained with only secondary antibody as a negative experimental control. The intracellular Cat B expression in U87-MG cell line was verified before intracranially implanting tumor cells into animals.

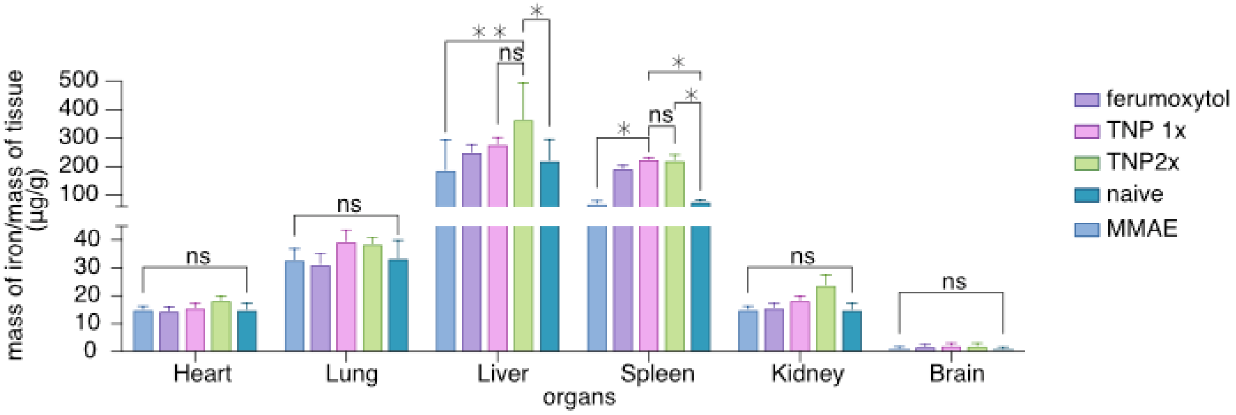

Figure S6. Biodistribution of TNPs. Mice (n = 3) were intravenously injected with TNP (0.3 mg/Kg of MMAE, 25 mg/Kg of Fe) once a week (TNP1×) or twice a week (TNP2×), MMAE (0.3 mg/Kg), ferumoxytol (25 mg/Kg), or PBS control. Organs were collected and digested for ICP-OES analysis.

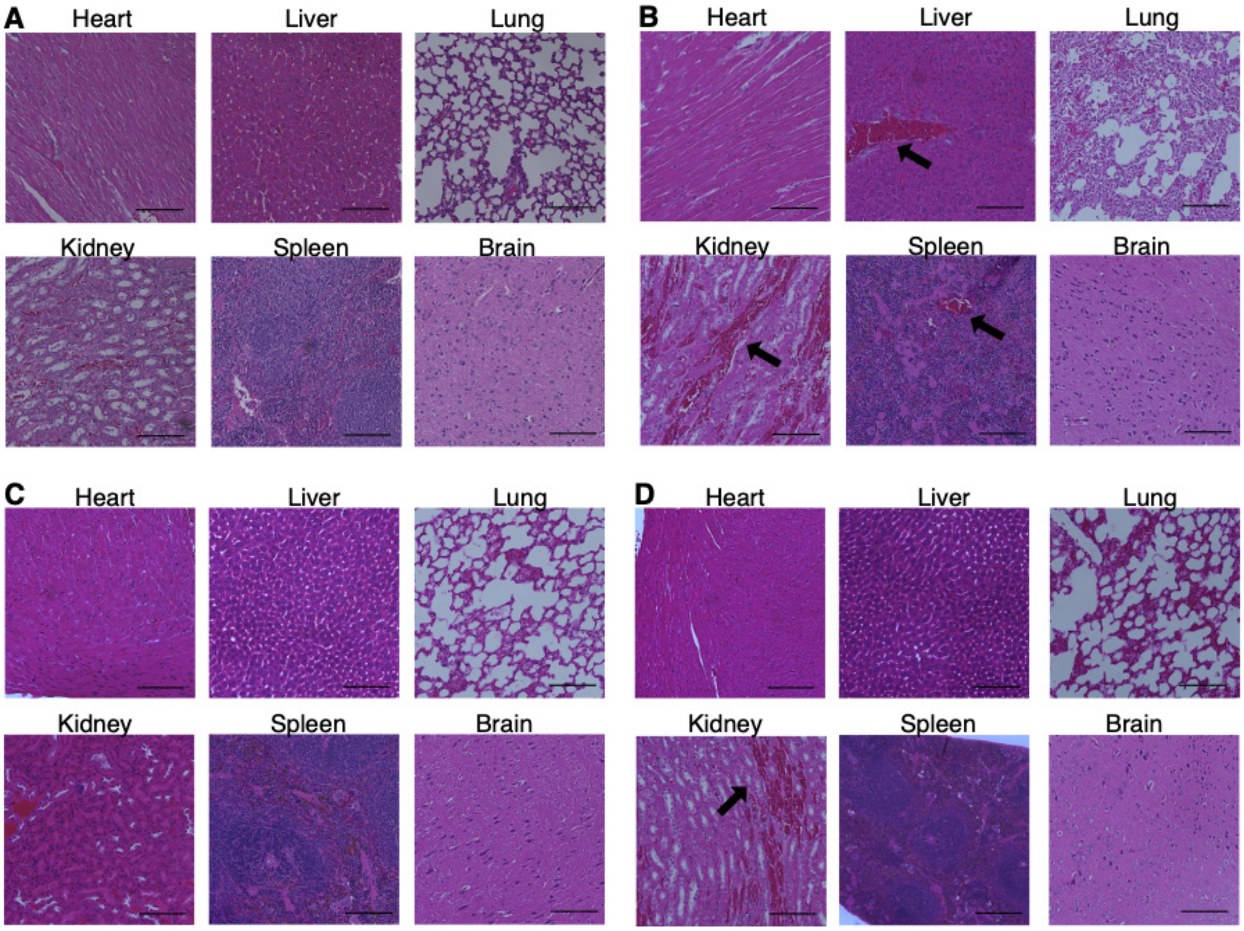

Figure S7. Representative H&E images of harvested organs (A) Control mice; (B)MMAE-treated; TNP (0.3 mg/Kg of MMAE, 25 mg/Kg of Fe) (C) once a week (TNP1×) or (D) twice a week (TNP2×). Images show significant necrosis in different organs in MMAE-treated mice as well as TNP2×-treated mice. Black arrows indicate necrosis. Organs from TNP1×-treated mice appear normal. Scale bars indicate 120 μm.

Detection of iron using Prussian Blue staining in the harvested brain confirmed accumulation of TNPs in tumors for TNP-treated and TNP+radiation treated groups (**Figure S7**).

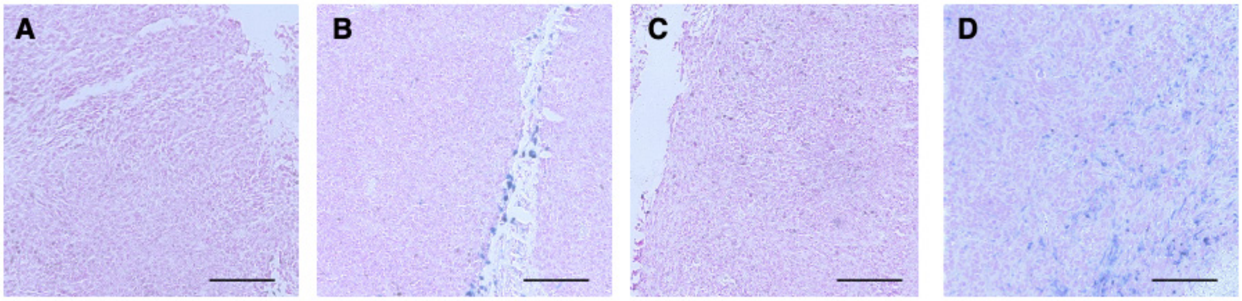

Figure S8. TNPs are delivered to GBM tumors. Representative images of cleaved Prussian Blue staining of mouse brains treated with (A) control; (B) TNP; (C) radiation, or (D) TNP+radiation. Blue color indicates iron positive staining. Nuclei counterstained with fast red. Scale bars are 100 µm.

In harvested murine tumor tissues, GBM treated with TNP or combination of TNP+radiation demonstrated higher apoptosis levels compared to PBS-treated controls (**Figure S9**).

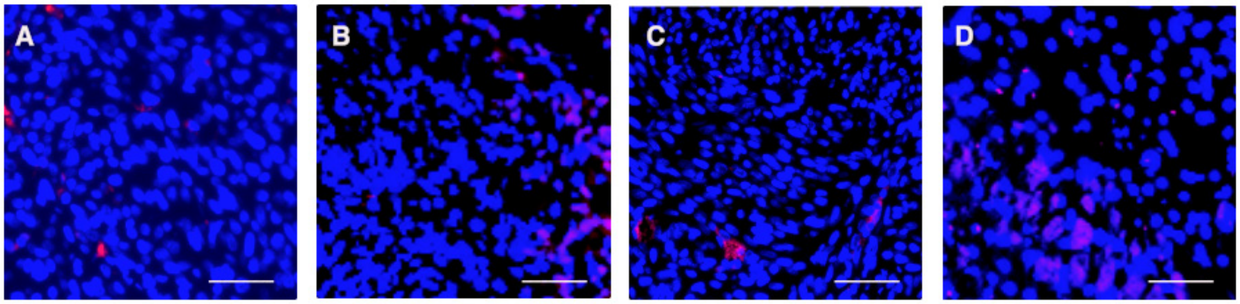

Figure S9. TNPs are delivered to GBM tumors and induce tumor apoptosis. Representative immunofluorescence images for cleaved caspase-3 in (A) control-; (B) TNP-; (C) radiation; (D) TNP+radiation treated GBM tumors. Red indicates cleaved caspase-3–positive cells. Nuclei counterstained with DAPI. Scale bars are 100 μm.

The mouse brains were collected after TNP treatment and embedded in paraffin. After dehydration and hydration, brain tissue slices were stained with cleaved caspase-3 to detect apoptotic cells. Images indicate multiple apoptotic cells in GBM while no or sporadic apoptotic cells were found in normal brain (**Figure S10**).

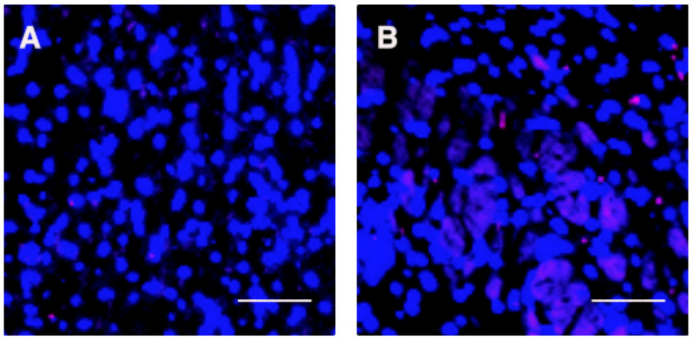

Figure S10. TNPs induce apoptosis in GBM but not normal brain. Representative immunofluorescence images for cleaved caspase-3 in (A) normal brain and (B) GBM. Red indicates cleaved caspase-3–positive cells. Nuclei were counterstained with DAPI. Scale bars are 100 μm.
